# Supplementary material for: The brain of fetuses with congenital diaphragmatic hernia shows signs of hypoxic injury with loss of progenitor cells, neurons, and oligodendrocytes
Source: Sci Rep. 2024 Jun 13;14:13680. doi: 10.1038/s41598-024-64412-x (PMC11176194; doi:10.1038/s41598-024-64412-x)
Supplement: Supplementary file 1 — Supplementary Information 1. [file 41598_2024_64412_MOESM1_ESM.pdf]

Supplementary figure 1

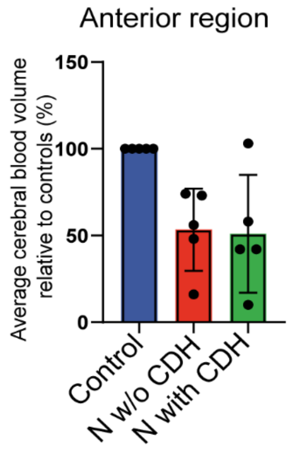

Supplementary Fig. 1 Cerebral perfusion quantification of the anterior brain region in CDH fetal rats. No differences in cerebral perfusion were observed among three experimental groups. Data is presented as mean ± SD.

Supplementary figure 2

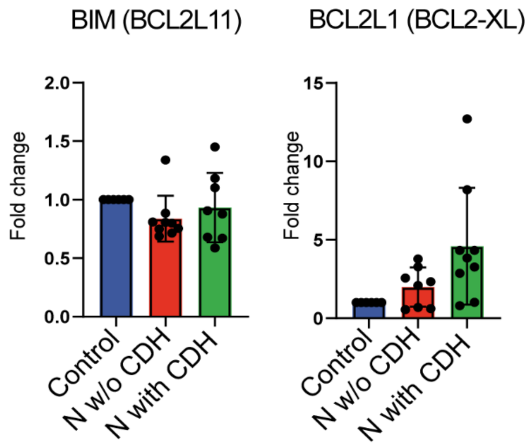

Supplementary Fig 2 Gene expression of anti-apoptotic marker. RT-qPCR experiments show no differences in gene expression of anti-apoptotic *BCL2L11* or *BCL2-XL* in the brain of pups among the three experimental groups. Control (n=6), N w/o CDH (n=8), N with CDH (n=7). Data are presented as mean ± SD.

Supplementary figure 3

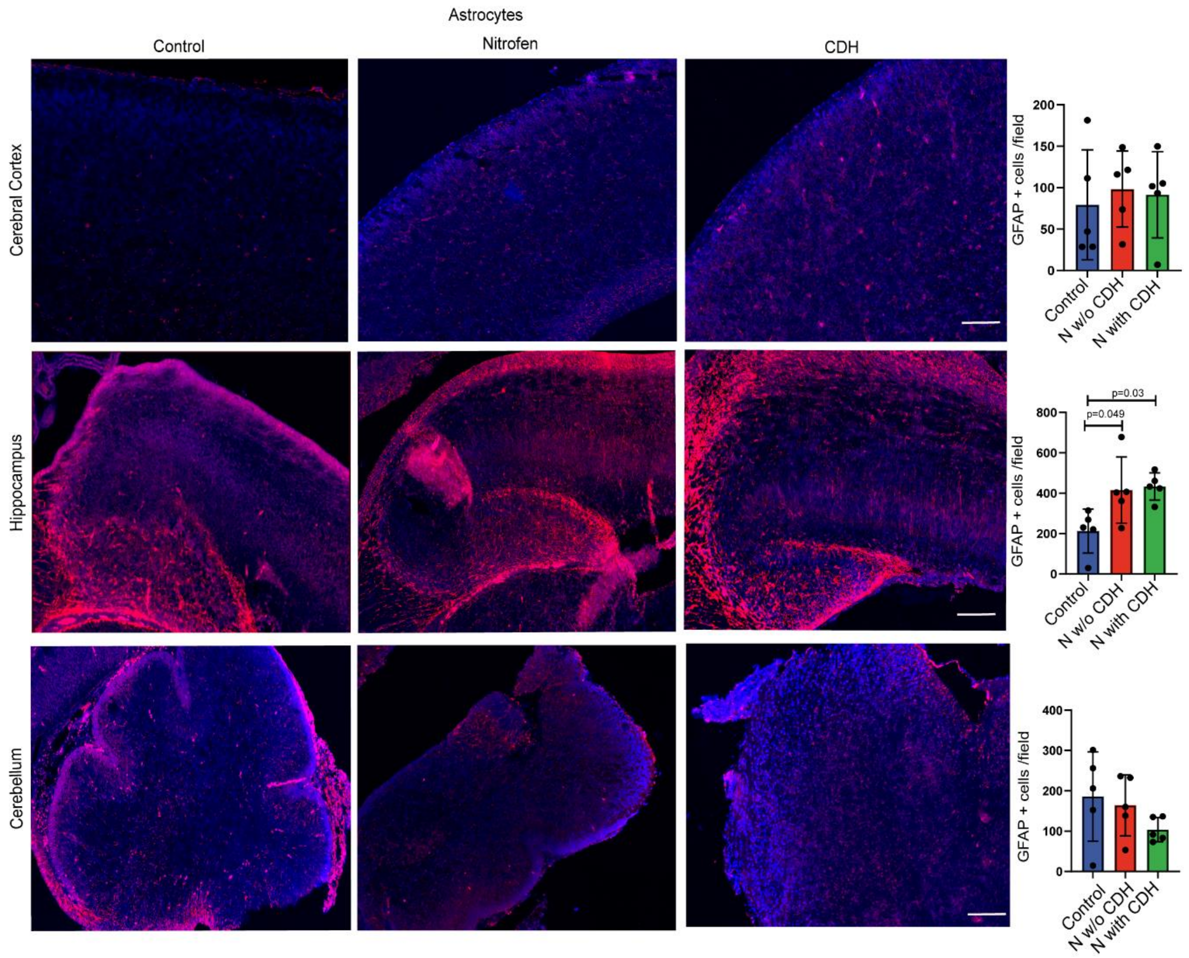

Supplementary Fig. 3 Astrocyte activation in the CDH rat fetal brain. Representative immunofluorescence images of astrocyte marker glial fibrillary acidic protein (GFAP, red) and nuclear marker (DAPI, blue) in the cerebral cortex, hippocampus, and cerebellum. Nitrofen-exposure alone caused an increase in astrocyte activation in the hippocampus. Control (n=5), N w/o CDH (n=5), N with CDH (n=5). Data are presented as mean ± SD. Scale bar = 50 μm.

Supplementary figure 4

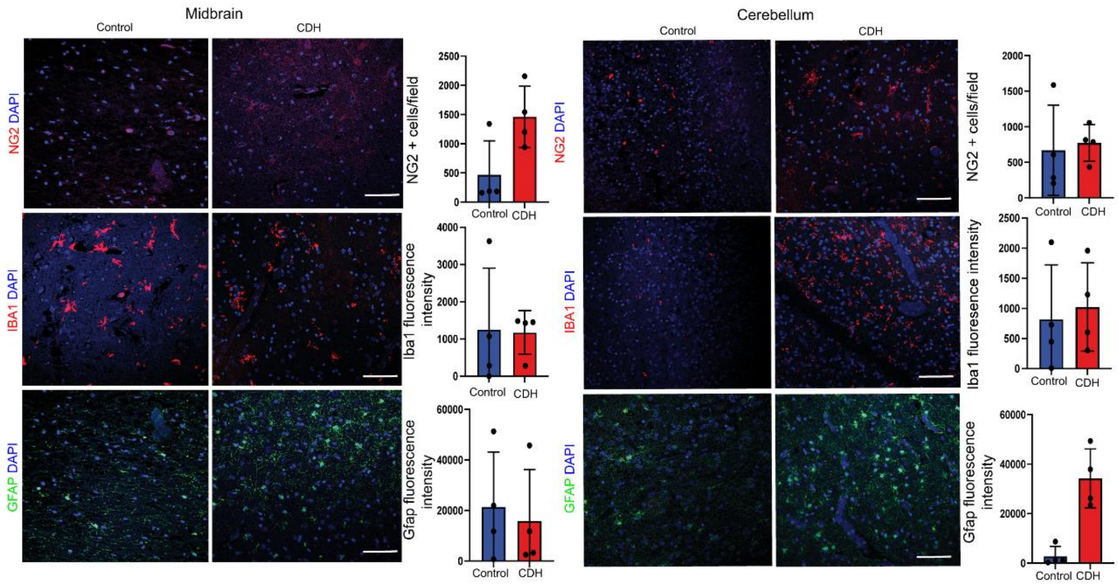

Supplementary Fig. 4. Oligodendrocyte progenitor cells, microglia, and astrocytes in the midbrain and cerebellum/pons of human fetuses. Representative immunofluorescence images of oligodendrocyte progenitor cells (NG2+) activated microglia (Iba1+), and astrocytes (GFAP+), quantified by number of cells per field (NG2) or fluorescence intensity (Iba1, GFAP).

Supplementary figure 5

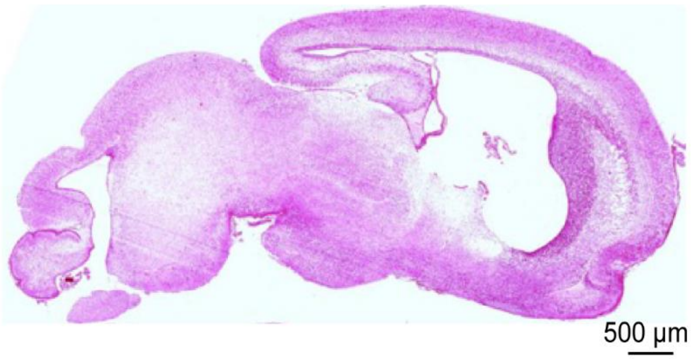

Supplementary Fig. 5. Representative image of Hematoxylin and Eosin stained sagittal/medial brain section of E21.5 rat pup at the specific histological level used for the immunofluorescence experiments.
