# Supplementary material for: The brain of fetuses with congenital diaphragmatic hernia shows signs of hypoxic injury with loss of progenitor cells, neurons, and oligodendrocytes
Source: Sci Rep. 2024 Jun 13;14:13680. doi: 10.1038/s41598-024-64412-x (PMC11176194; doi:10.1038/s41598-024-64412-x)
Supplement: Supplementary file 2 — Supplementary Information 2. [file 41598_2024_64412_MOESM2_ESM.pdf]

Supplementary Table 1. Primer sequences

Gene	Forward primer	Reverse primer
hif1α	AAGTGGCAACTGATGAGCAA	GGCGAGAACGAGAAGAAAAA
Glut1	AGAGAGACCAAAGCGTGGTG	GCAGTTCGGCTATAACACTGG
Bax	AGCAAACTGGTGCTCAAGGC	CCACAAAGATGGTCACTGTC
CASP9	AGTTCCCGGGTGCTGTCTAT	GCCATGGTCTTTCTGCTCAC
BCL2L11	TGAGTGTGACAGAGAAGGTGG	CCTTATGGAAGCTTGCGATTCCG
BCL2L1	GCCGTCGAGCCGATGAAATA	GGTTGGCAAGGCGTGATCTA
BIP	GGCGTGAGGTAGAAAAGG	ATGGTAGAGCGGAACAGG
EIF2S1	AACAAATGGAGAAAGTGCTG	ACCAGTCCCAAAGTCAAAC
ATF6	GGTTCTGTCTTCCACTCCA	GGCTTCCTGTCTCCTTCA
GAPDH	CCCATCACCATCTTCCAGGAGCGAG	GTTGTCATGGATGACCTTGGCCAGG

Supplementary Table 2. Primary & Secondary antibodies

Primary antibody	Marker	Species	Assay & Dilution	Company	Catalog number
HIF1α	Hypoxia	mouse	WB: 1:1000<br>IF: 1:100	Novus	NB100-105
Glut1	Hypoxia	rabbit	WB: 1:1000	Santa Cruz	sc-7903
Histone 3	Loading control	rabbit	WB:1000	Cell signaling	9715S
Bax	Pro-apoptosis	rabbit	WB: 1:1000	Cell Signaling	2772
CC9	Pro-apoptosis	rabbit	WB: 1:1000	Cell Signaling	9509P
BiP	ER stress	rabbit	WB: 1:1000	Cell Signaling	3177T
p-eIF2α	ER stress	rabbit	WB: 1:1000	Cell signaling	9721S
ATF6	ER stress	mouse	WB: 1:1000	Novus	NBP1-40256SS
NeuN	Mature neurons	mouse	IF: 1:200	EMD Millipore Sigma	MAB377
TMEM119	Microglia	rabbit	IF: 1:100	abcam	ab185337
Ki67	Proliferation	rat	IF 1:200	Novus Biologicals	NB500-170
Olig2	Oligodendrocytes (progenitor and mature)	mouse	IF: 1:50	R and D systems	AF2418

NG2	Oligodendrocyte progenitor cell	rabbit	IF: 1:200	abcam	ab275024
MBP	Mylein basic protein	rabbit	IF: 1:200	EDM millipore Sigma	M3821
GFAP	Astrocytes	chicken	IF: 1:500	abcam	ab4674
