# Supplementary material for: The brain of fetuses with congenital diaphragmatic hernia shows signs of hypoxic injury with loss of progenitor cells, neurons, and oligodendrocytes
Source: Sci Rep. 2024 Jun 13;14:13680. doi: 10.1038/s41598-024-64412-x (PMC11176194; doi:10.1038/s41598-024-64412-x)

Full unedited gel for Figure 1b

HIF1α

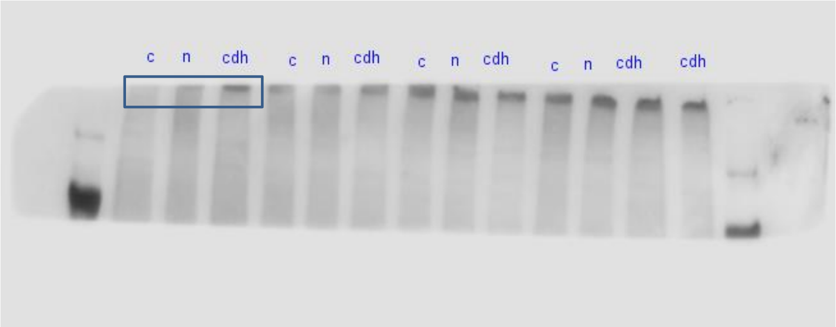

Glut1

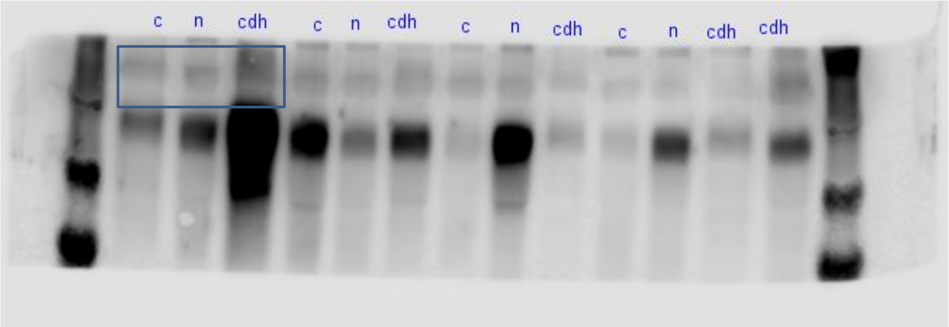

H3

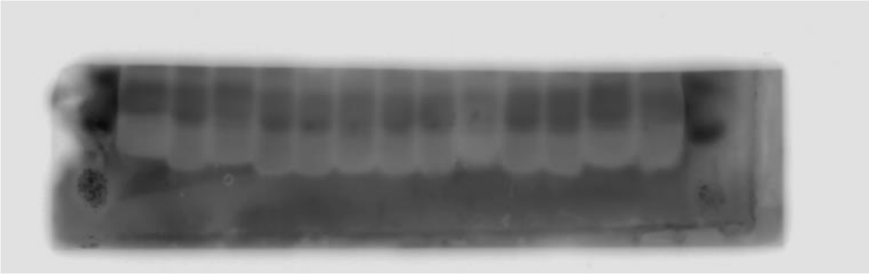

Full unedited gel for Figure 2a

CC9

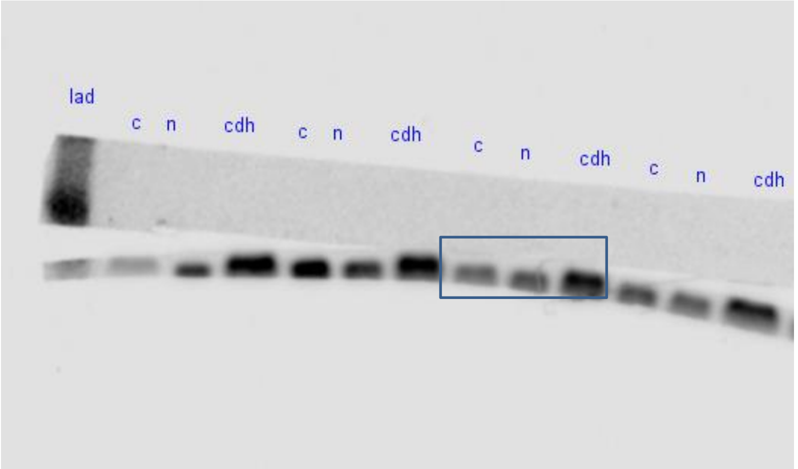

Bax

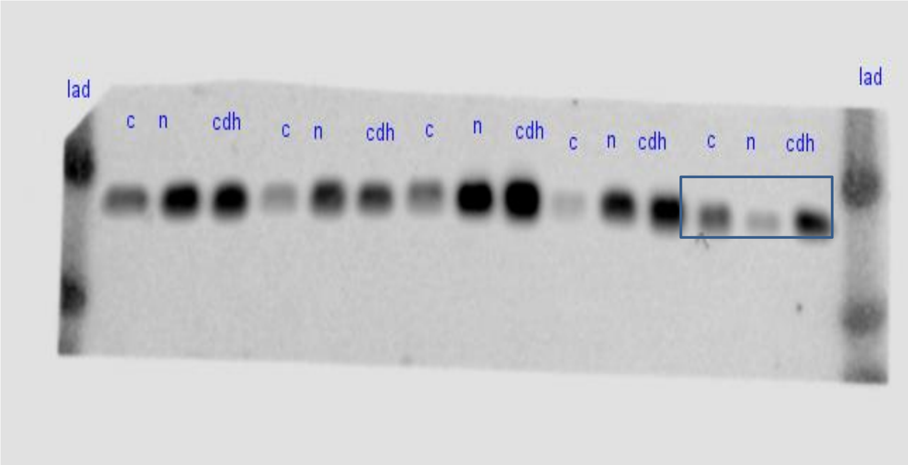

H3

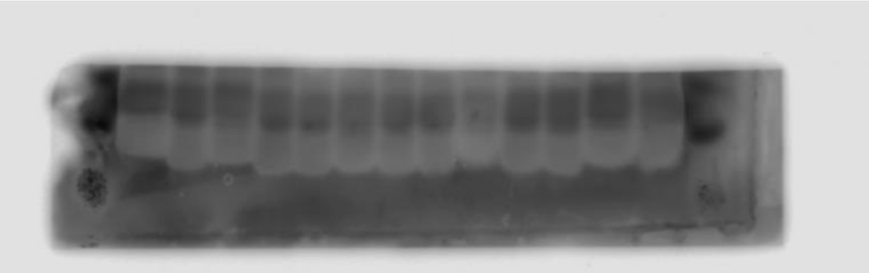

Full unedited gel for Figure 2e

Atf6

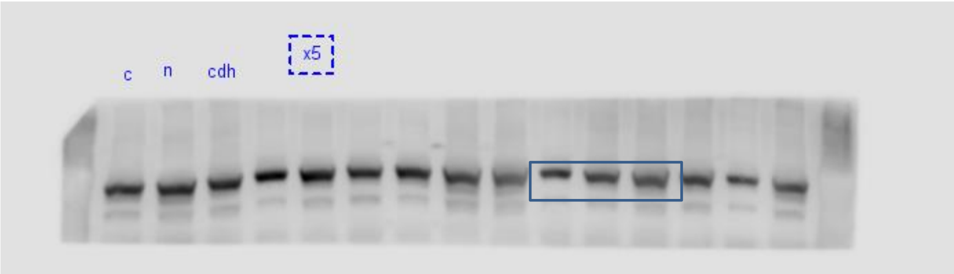

Bip

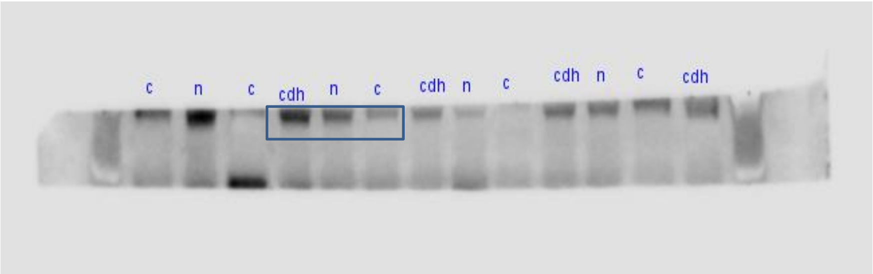

pEIF2α

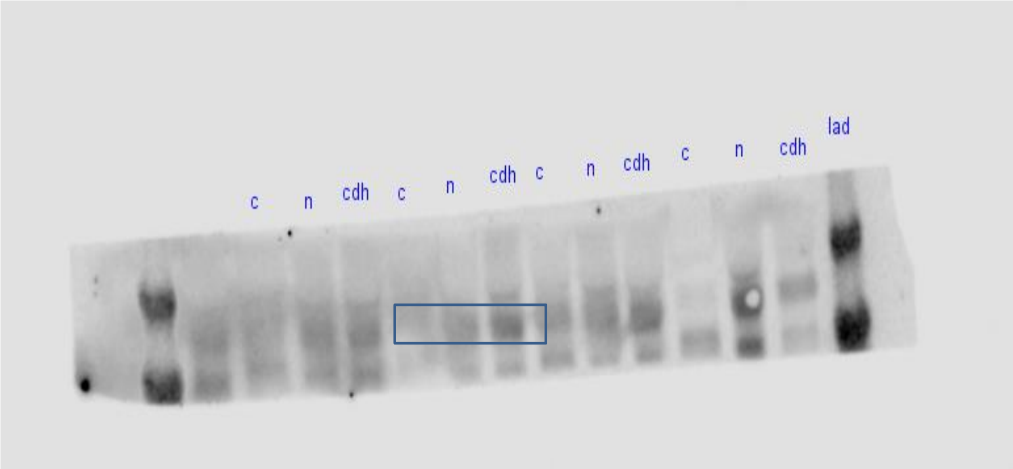

B-actin

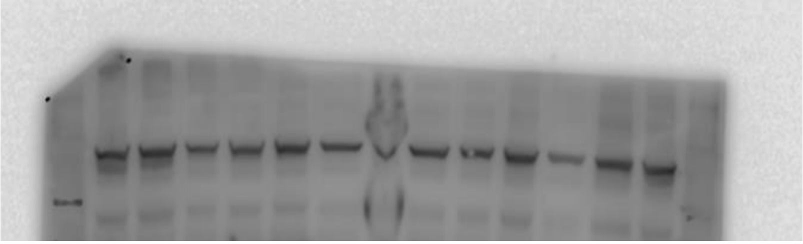

H3

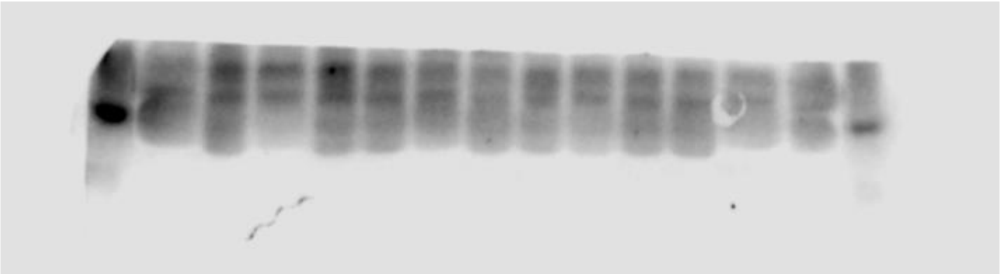

Full unedited gel for Figure 5a

spc

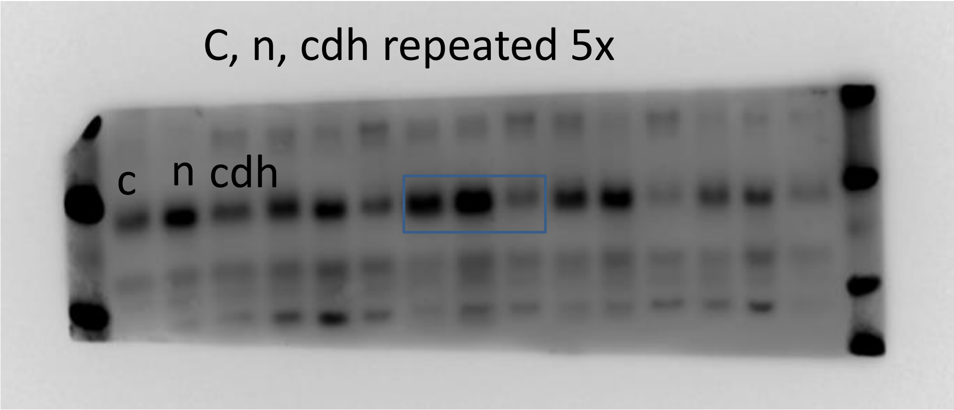

B-actin

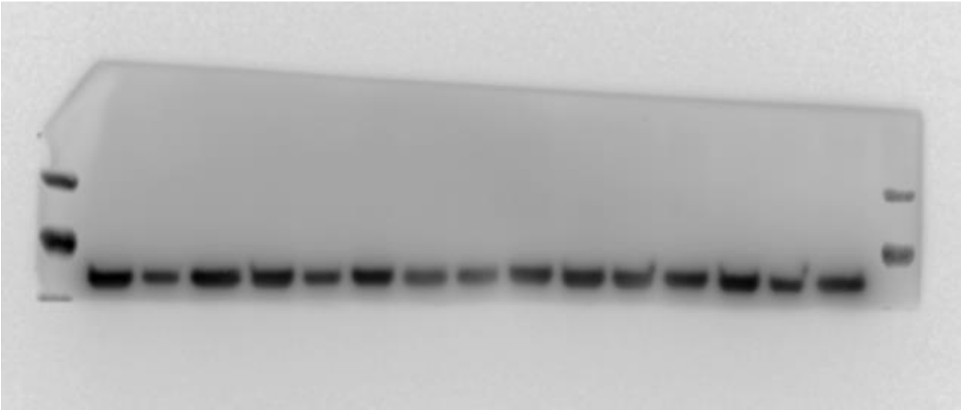

Supplement: Supplementary file 3 — Supplementary Information 3. [file 41598_2024_64412_MOESM3_ESM.pdf]
